# Ophthalmology

**Published:** 1899-12

**Authors:** Alvin A. Hubbell

**Affiliations:** Buffalo, N. Y., Clinical professor of ophthalmology in the University of Buffalo


### Progress in Medical Science.

### OPHTHALMOLOGY.

Conducted by ALVIN A. HUBBELL, M. D., Buffalo, N. Y.,
Clinical professor of ophthalmology in the University of Buffalo.

### THE EYE AFFECTED BY PATHOLOGICAL CONDITIONS OF THE TEETH.

Lagleyze (*Archives D'Ophthalmologie*, March, 1899; *Brooklyn Med. Journal*, September, 1899) claims that the following ocular affections may sometimes be of dental origin: Epiphora, blepharitis, conjunctivitis, keratitis, glaucoma, spasm of accommodation, mydriasis, blepharospasm, strabismus, neuralgia, photophobia and amblyopia. Caries or periostitis of upper molars is not an infrequent cause of a diseased condition of the eye. Hypersecretion of the lachrymal gland is often of reflex origin, the dental irritation being communicated by filament of the orbital branch of the superior maxillary division of the trigeminus.

Many cases have been published which show the possibility of glaucoma being a reflex affection. Mention is made of a case in Abadie's clinic; sclerotomy had been done twice without any beneficial results. However, upon the extraction of a diseased tooth, the intraocular tension was quickly relieved. Paralysis or paresis or accommodation may be caused by odontalgia. It is a common observation that detail neuralgias cause a hyperesthetic condition of the eye. Galezowski says that monocular mydriasis, eight times out of ten, is due to dental affections. However, Lagleyze regards this statement as an exaggeration. Redard, Mitchell and others cite cases of strasbismus occurring in infants during the period of dentition.

Vasomotor paralysis, especially in children, causes a congestion of the conjunctiva and lessens its resisting power, and, as a result, the eye is more susceptible to the action of microörganisms. Power reports a case of ulcer of the cornea and anesthesia of the parts supplied by the ophthalmic branch. After extraction of diseased teeth the corneal trouble was much improved and the anesthesia entirely disappeared. Abadie is of the opinion that stricture of the lachrymal duct is very frequently caused by a periostitis extending slowly up the superior maxilla. It is considerd advisable to carefully examine the teeth in all ocular diseases which are of doubtful etiology and which do not readily yield to the ordinary methods of treatment.

COMPLICATIONS FOLLOWING THE ENUCLEATION OF THE EYEBALL.

IN THE last Annual Report, 1898, of the *Transactions of the Ophthalmological Society of the United Kingdom* (*Brooklyn Med. Journal*, September, 1899,) an exceedingly interesting report was submitted by a committee appointed to consider the relative value of simple excision of the eyeball and the operations which have been substituted therefore. One of the questions considered was the frequency of meningitis after excision. Reports from a number of large hospitals show that out of nearly 11,000 excisions there were only 7 cases of meningitis. The committee regarded it as highly probable that in a certain proportion of the cases in which the patients have died from meningitis after excision of a suppurating eye, the meningitis began before the operation, and that the patients would have died had no operation been performed. Among some of the advantages of Mule's operation may be mentioned that in quite a number of cases there was excessive reaction, sloughing of the sclerotic and subsequent pain and irritation in the stump.

